# Study of the dynamic expression of Meis1 in mice 

**Published:** 2013-02

**Authors:** Li Hai-Xia, Guo Xin-Yu, Xie Yan, Yuan Qi-Long, Ge Ming-Xiao, Zhang Jin-Yu

**Affiliations:** *Reproductive Medicine Center, Department of Obstetrics and Gynecology, Guangzhou General Hospital of Guangzhou Military Region, Guangzhou 510010, Guangdong, China.*

**Keywords:** *Meis1*, *Embryonic implantation*, *Endometrial receptivity*, *Peri-implantation*

## Abstract

**Background: **Aggressive embryo and receptive endometrium are necessary for successful implantation. On this time endometrium transformates to receptive state, which permits embryonic implantation. Studies about embryonic implantation and endometrial receptivity are always a hot spot in the field of reproductive medicine.

**Objective:** To investigate the expression pattern of Meis1 during peri-implantation in mice endometrium.

**Materials and Methods:** Mice for experiment were raised in SPF environment. The mice were mated with a female/male ratio of 2:1. The female mice with detected plugs were regarded as pregnant day 1 (pd1). Endometrial tissues were collected respectively on pd1, pd2, pd4, pd5 and pd6. Immunohistochemistry was used to detect the location of Meis1 in mice endometrium. The expression level of mRNA and protein of Meis1 were further detected using Quantitative PCR and Western blotting, respectively.

**Results:** We found that Meis1 is located in the cytoplasm and membrane of endometrial glandual epithelium cells and the nucleus of endometrial stromal and decidual cells. Both Quantitative RT-PCR and western blotting showed that Meis1 expressed regularly in mice endometrium. Meis1 mRNA expressed weakly on pd1, then significantly increased on pd4 (p=0.018), and achieved to a peak on pd5 (p=0.0012), it showed a decrease trend on pd6. Meis1 protein expressed weakly on pd1 and pd2, then significantly increased on pd4 and pd5 (p=0.0019), it showed a decrease trend on pd6

**Conclusion:** Meis1 is dynamically expressed in mice endometrium during peri-implantation. The time that Meis1 expression reaches its peak value is coincident with the implantation window, which implied that Meis1 is closely related with embryonic implantation.

## Introduction

Embryonic implantation is one of the key steps in mammal reproduction. There are many conditions that must be satisfied in order for a successful implantation to take place, once there is something wrong, it will most likely to be a miscarriage (1).

Homeobox genes (HOX) are a group of gene family that are involved in the regulation of morphogenesis, and are highly conservative among different species, including animals, fungi and plants. HOX regulates embryo development and cell differentiation through forming positive and negative regulation feedbacks with upstream signaling molecules and downstream target genes (2). It was reported that HOXA10 is involved in development of female genital system; it has a key role to uterus development, endometrial proliferation and differentiation, as well as endometrial receptivity establishment (3). 

HOX genes bind to regulatory DNA sequence of downstream target gene through coiling-turn-coiling motif so as to activate or inhibit the expression of downstream target genes and the capability of HOX genes to discriminate target genes is restrict (4). Meis1 (Myeloid ecotropic viral integration site) is a member of Three Amino Acid Loop Extension (TALE) family, which locates on 2p13-p14 of human chromosome and end of mouse chromosome11. 

Hetero-dimers comprised of the HOX protein and Meis will greatly reinforce the affinity and specificity of HOX’s ability to bind target sequence (5). For example, the Meis1-HOXA10 hetero-dimer specifically identifies DNA sequence of TGACAGTTAT (4).

Receptive endometrium is necessary for successful implantation, and increasing studies have indicated that HOXA10 and Meis1 are intimately correlated with the establishment of endometrial receptivity (6). It was reported that Meis1 participates in the process of embryonic implantation as a co-factor of HOXA10 (7). 

In this study, to investigate the association between Meis1 and endometrial receptivity, we detected the dynamic expression pattern of Meis1 in mice endometrium during peri-implantation.

## Materials and methods

**Animals**

This is a descriptive study. The study was approved by the Animal Care and Use Committee (ACUC) of Guangzhou General Hospital of Guangzhou Military Region. Virgin six to eight-week-old specific pathogen free KM mice were supplied by Animals Experimental Center in Guangzhou General Hospital of Guangzhou Military Region. The mice were housed in 12/12h light/dark cycle at 25±0.5^o^C and 50-60% humidity and were fed ad libitum with a standard pellet diet and water. 

The mice were mated when they achieved to 18-20-week-old or 20-25g body weight, The mice were put together with a female/male ratio of 2:1 at 5.00 pm. the plugs were detected at 8.00 am. the next day, Once the plug appeared, it was regarded as pregnant day 1(pd1). All pregnant mice were divided into five groups according to their pregnant duration (pd1, pd2, pd4, pd5 and pd6), each group contains 10 mice. 

**Tissue collection**

Endometrial tissue was collected routinely, endometrial biopsy were done at 9.00 am every day. The endometrial tissue was washed in frozen PBS. Half of the tissue sample was immediately frozen in nitrogen until the extraction of mRNA and protein. The other tissues were fixed in 10% formalin, embedded in paraffin, and then sectioned.

**Immunohistochemistry**

The expression of mouse Meis1 was evaluated by immunohistochemistry using a rabbit polyclonal antibody to Meis1 (ab19867，Abcam, USA). The above sections were deparaffinized in xylene and gradient ethanol. Endogenous peroxidase was blocked by 3% H_2_O_2_ at room temperature for 10 min. After blocking for 20 min with 1.5% normal goat serum in PBS, the sections were incubated overnight at 4^o^C with primary Meis1 antibody (1:100) in PBS. 

The sections were then incubated with biotinylated goat anti-rabbit IgG (ZDR-5306, Zhongshan Goldenbrioge Biotechnology co. LTD, CHINA) as a secondary antibody at 1:50 and incubated at 37^o^C for 20 min, then with DAB (400 mg/ml) at room temperature for 5 min, counterstained by haematoxylin, following dehydration, and clearing. Finally mounting was done for observation with microscope. Negative control was set for each group.

**RNA isolation and Quantitative RT-PCR**

Total RNA of each sample was extracted routinely using the total RNA isolation reagent (Invitrogen) according to the manufacturer’s instructions. Reverse transcription was performed with the M-MLV Reverse Transcriptase (Promega) using 1μl of total RNA. The cDNA was sent for Quantitative PCR reactions, which were performed using the ABI 7700 system and the amplifications were done using the SYBR Green PCR Master Mix (Applied Biosystems). The primers for mouse Meis1 were: forward primer 5’-AAG CAG TTG GCA CAA GAT ACA GGA C-3’, reverse primer 5’-TGA CTG CTC GGT TGG ACT GG-3’, and for mouse β-actin: forward primer 5’-GAG ACC TTC AAC ACC CCA GCC-3’, reverse primer 5’-TCG GGG CAT CGG AAC CGC TCA-3’. 

The thermal cycling conditions were composed of 50ºC for 2 min followed by an initial denaturation step at 95ºC for 10 min, 30 cycles at 95ºC for 40s, 50ºC for 50s and 72ºC for 60s. The experiments were carried out in duplicate for each data point. The relative quantification in gene expression was determined using the 2^ΔΔCt^ method. Using this method, we obtained the fold changes in gene expression normalized to β-actin.

**Western blotting**

Tissues from each mouse uterus were washed twice with PBS and lysed on ice in lysis buffer. Solid cellular debris was removed by centrifugation at 12,000 rpm for 5min. Protein concentration was measured by the BCA methods. The protein samples (50μg each) were subjected to 10% SDS-PAGE and transferred onto PVDF using a Bio-Rad electroblot apparatus (Bio-Rad). Non-specific binding sites were blocked in 5% (w/v) non-fat milk in TBS containing 0.05% (v/v) Tween-20. The primary antibody was added and incubated at 4^o^C overnight. 

The dilutions for rabbit anti-Meis1 (Abcam) and rabbit anti-actin antibody (Santa Cruz) were 1:200 and 1:500, respectively. After washing by TBS, the secondary antibody goat peroxidase-conjugated anti-rabbit IgG (Zhongshan Goldenbrioge Biotechnology) was used at a dilution of 1: 1000, and incubated at 37^o^C for 1h. After washing by TBS, protein bands were visualized by ECL (Pierce Biotechnology). Densitometric analysis of the film was performed using a Model GS-710 imaging densitometer (Bio-Rad Laboratories) in transmittance mode and analyzed using Bio-Rad Discovery software.

**Statistical analysis**

All numerical results are expressed as the mean+SD. The Meis1 expression in endometrium during peri-implantation were analyzed by analysis of variance. P<0.05 was considered significant in all tests. The Statistical Package for the Social Sciences software package for Windows (Version 13.0) was used for all statistical analyses. 

## Results

**Immunohistochemical localization of Meis1 duiring the implantation window of mice**

Meis1 expressed in grandular epithelial cells, endometrial stromal cells and decidual cells. With the elongation of pregnancy duration, the expression of Meis1 gradually increased. Different cell types showed different expression pattern. Grandular epithelial cells showed a cytoplasm and membrane expression pattern while the endometrial stromal cells and decidual cells showed nucleus expression. It was weakly expressed both in grandular epithelial cells and in endometrial stromal cells on pd1, then mildly increased on pd2, it significantly increased on pd4, especially in nucleus of endometrial stromal cells, and achieved a peak on pd5. In decidual tissue, Meis1 located in nucleus. 

**Expression of Meis1 mRNA during peri-implantation**

Meis1 mRNA was expressed in mice endometrium during the peri-implantation, its expression levels were gradually increased, and achieved the peak on pd5, then decreased on pd6.

**Expression of Meis1 protein during peri-implantation**

The expression of endometrial Meis1 during peri-implantation was further comfirmed by Western blotting (Figure 3). When normalized with β-actin expression levels, Meis1 protein was also significantly increased on pd4 (p<0.01), and achieved the peak on pd5, then decreased on pd6. 

**Figure1:**
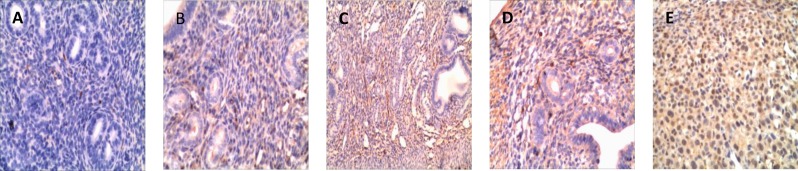
Immunohistochemical staining for the expression of endometrial Meis1 during peri-implantation. A～E were the expression of Meis1 in the mouse endometrium of pregnant day 1, day 2, day 4, day 5 and day 6, respectively. Photomicrographs were taken at ×200 magnification

**Figure 2:**
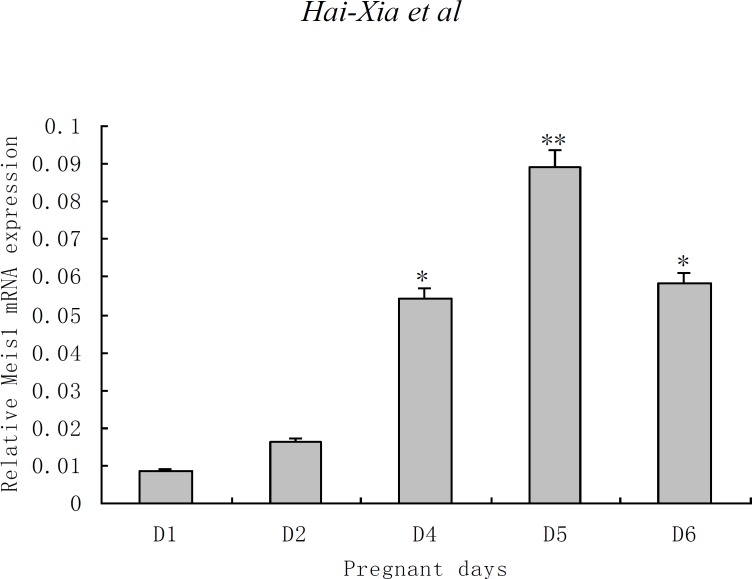
Meis1 mRNA expressioin in the peri-implantation endometrium of early pregnant mouse. *p<0.05, compared with pd1; **p<0.01, compared with pd1

**Figure 3:**
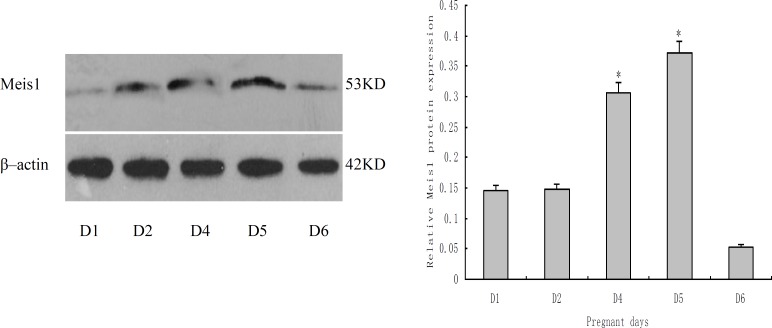
Meis1 protein expression in peri-implantation endometrium of early pregnant mouse. *p<0.01, compared with pd1

## Discussion

Embryonic implantation is a complex biological process between maternal endometrium and embryo, which includes blastocystic localization, adhesion, invasion and endometrial decidualization, etc. (1, 8). The process of implantation involves a complicate succession of genetic and cellular interactions, all of which must be executed within an optimal time frame. The peri-implantation is started by preparations in the endometrium of the uterus, and the endometrium transforms to a receptive state. It was reported that Meis1 is intimately correlated with the establishment of endometrial receptivity (6, 7). 

Therefore we investigate the expression pattern of Meis1 in mice endometrium during peri-implantation. We detected the expression of Meis1 during various time of peri-implantation in mice endometrium using different methods. Our data provides evidence that Meis1 is dynamically regulated during peri-implantation, which shows it involved in implantation. We found that the Meis1 expression gradually increased along with the pregnancy duration, and reached a peak on pd5, and then it decreased, which is coincident with the implantation window. 

The TALE family of homeodomain proteins represents a most important group of HOX cofactors whom shares sequence homology outside of the homeobox domain. Single TALE proteins have only low DNA-binding activity and specificity, however, the unique TALE motif within their homeodomain allows TALE proteins to interact with other homeobox-containing proteins. 

Once combined with another subfamily of HOX-class homeobox protein, they show powerful and specific downstream target promoter regulation. Meis1 was first identified as a major integration site for leukemogenic virus in a murine leukemia model, aberrant activation of Meis1 is related to the genesis of human leukemia, and the defect of Meis1 is intimately related to congenital heart disease as well (9, 10). Orlovsky *et al* stated that down-regulation of homeobox genes Meis1 and HOXA in MLL-rearranged acute leukemia impairs engraftment and reduces proliferation (11).

HOXA10 is essential for normal embryonic uterine development. Benson *et al* induced altered HOXA10 expression as a result of targeted mutation, which leads to abnormal uterine development (12). It was reported that the Hox cofactors Meis1 and Pbx act upstream of gata1 to regulate primitive hematopoiesis (13). Pbx and Meis cofactors are also involved in HOXA10 target gene recognition in the endometrial cells, which suggests that these proteins may be essential for endometrial receptivity. The temporal and spatial expression patterns of Meis1 and Pbx2 as well as that of HOXA10 indicated that HOX-Pbx2 dimers in the glands and HOXA10-Pbx2-Meis1 trimers in the stroma activate or repress downstream target gene expression. These transcription factor complexes likely regulate endometrial receptivity (14). 

It was confirmed that HOX cofactors expression in normal human ovary is temporally and spatially specific and regulated by FSH and GDF-9 in granulose cells (15). HOX proteins require co-operation with other proteins to bind the target DNA. Meis1 in particular aids 5’ HOX proteins, like HOXA10, to gain this specificity. Meis1 physically interacts with 5’ HOX proteins HOXA9-11 by forming heterodimeric binding complexes on a DNA target containing a MEIS1 site (TGA CAG) and an Abd-B-like Hox site (TTT TAC GAC). Previous studies confirmed that Meis1 gene and HOXA9-13 genes are co-expressed throughout Müllerian duct differentiation, which suggests that Meis1 plays an important role in embryonic female genital tract development (16). The temporal and spatital expression pattern of Meis1 and HOXA10 indicate that HOXA10- Meis1 dimers activate or repress downstream target gene expression.

Previous studies confirmed that the expression of HOXA10 in endometrium is regulated by ovarian estrogen and progestogen. Both hormones can enhance the expression of HOXA10, and the effect of the latter is more obviously; The HOXA10 expression in endometrial stromal cells is intensified by combination of estrogen and progestogen (17). It was confirmed that Meis1 expressed both in endometrial stromal cells and in glandular epithelial cells, and it is expressed stronger in the latter. 

The expression pattern of Meis1gene was investigated at different stages of human menstrual cycle, which demonstrated that Meis1 is expressed in endometrium at different levels, depending on the menstrual cycle stage. Meis1 mRNA level is significantly increased in mid-secretory phase, closely resembling the expression pattern of HOXA10. It implied that Meis1 may be one of the regulating factors of endometrial receptivity formation in implantation period, and it was important for the process of embryo implantation mediated by HOXA10 (7). The immunohistochemical research showed that Meis1 was widely expressed in mouse uterine issue during the peri-implantation period, while different cells showed a different expression profile. Meis1 was expressed in the cytoplasm and membrane of grandular epithelial cells, while in the nucleus of endometrial stromal cells and decidual cells.

## Conclusion

Meis1 is dynamically expressed in mice endometrium during peri-implantation period. The time for Meis1 expression to reach its peak value is coincident with the implantation window. Our study indicates that Meis1 could be a potential molecular marker for endometrial receptivity.
